# Doxyl Nitroxide Spin Probes Can Modify Toxicity of Doxorubicin towards Fibroblast Cells

**DOI:** 10.3390/molecules25215138

**Published:** 2020-11-04

**Authors:** Jan Czepas, Karolina Matczak, Aneta Koceva-Chyła, Bartłomiej Grobelski, Zofia Jóźwiak, Krzysztof Gwoździński

**Affiliations:** 1 Department of Molecular Biophysics, Faculty of Biology and Environmental Protection, University of Łódź, 141/143 Pomorska st., 90-236 Łódź, Poland; bartlomiej.grobelski@umed.lodz.pl (B.G.); krzysztof.gwozdzinski@biol.uni.lodz.pl (K.G.); 2 Department of Medical Biophysics, Faculty of Biology and Environmental Protection, University of Łódź, 141/143 Pomorska st., 90-236 Łódź, Poland; karolina.matczak@biol.uni.lodz.pl (K.M.); aneta.koceva@biol.uni.lodz.pl (A.K.-C.); zofia.jozwiak@biol.uni.lodz.pl (Z.J.)

**Keywords:** doxyl nitroxide, α-Tocopherol, doxorubicin, cytotoxicity, anticancer activity, lipid peroxidation, membrane fluidity, apoptosis, electron paramagnetic resonance, nitroxide reduction, chemosensitizer

## Abstract

The biological properties of doxyl stearate nitroxides (DSs): 5-DS, Met-12-DS, and 16-DS, commonly used as spin probes, have not been explored in much detail so far. Furthermore, the influence of DSs on the cellular changes induced by the anticancer drug doxorubicin (DOX) has not yet been investigated. Therefore, we examined the cytotoxicity of DSs and their ability to induce cell death and to influence on fluidity and lipid peroxidation (LPO) in the plasma membrane of immortalised B14 fibroblasts, used as a model neoplastic cells, susceptible to DOX-induced changes. The influence of DSs on DOX toxicity was also investigated and compared with that of a natural reference antioxidant α-Tocopherol. By employing the trypan blue exclusion test and double fluorescent staining, we found a significant level of cytotoxicity for DSs and showed that their ability to induce apoptosis and modify plasma membrane fluidity (measured fluorimetrically) is more potent than for α-Tocopherol. The most cytotoxic nitroxide was 5-DS. The electron paramagnetic resonance (EPR) measurements revealed that 5-DS was reduced in B14 cells at the fastest and Met-12-DS at the slowest rate. In the presence of DOX, DSs were reduced slower than alone. The investigated compounds, administered with DOX, enhanced DOX-induced cell death and demonstrated concentration-dependent biphasic influence on membrane fluidity. A-Tocopherol showed weaker effects than DSs, regardless the mode of its application—alone or with DOX. High concentrations of α-Tocopherol and DSs decreased DOX-induced LPO. Substantial cytotoxicity of the DSs suggests that they should be used more carefully in the investigations performed on sensitive cells. Enhancement of DOX toxicity by DSs showed their potential to act as chemosensitizers of cancer cells to anthracycline chemotherapy.

## 1. Introduction

Doxorubicin (DOX) is an anthracycline anticancer drug commonly used in chemotherapy [1]. Biological and antitumour actions of DOX are multifactorial and involve its influence on different structures and processes in the cells [2]. DOX influence on cells starts at the sites of its interactions with components of the cellular membrane. Its binding to the cell membrane primarily depends on hydrophobic and electrostatic interactions with lipids, which determine, i.a., diffusion and efflux of the drug [3]. DOX evokes perturbations in cell membrane structure and, depending on concentration, causes an increase or a decrease in membrane fluidity [4,5]. It is also well known that changes in the cell membrane may affect cell functions and trigger cell death [6].

Doxorubicin binds to the plasma membrane but acts mostly inside the cell where the drug interacts with nuclear DNA. The intracellular concentration of DOX mainly depends on the rate of its passive diffusion and efflux, which determine the development of cellular resistance to this drug [7].

One of the main modes of anticancer action is determined by the production of cytotoxic reactive oxygen species (ROS) [8,9] in the cells in DOX reductive–oxidative cycle and in further reactions [2]. Among ROS, upon protonation, superoxide anion radicals initiate oxidation of fatty acid chains of cell membrane phospholipids [10], and particularly, hydroxyl radicals rapidly oxidize all biological molecules, including proteins and lipids [11]. Lipid peroxidation (LPO) is an undesired process, which causes changes and destabilisation of the structure of lipid membranes [12]. DOX has been shown to induce LPO as an early toxic effect [13]. LPO alters main cell functions based on membrane properties, such as transport and cell signalling, and intensely contributes to the occurrence and development of side-effects of DOX, such as cardiotoxicity and hepatotoxicity [1,14].

Doxorubicin-induced severe side-effects impose limitations in administering a drug dose [15]. Moreover, drug resistance of cancer cells can be developed [16]. Each of these factors seriously decreases efficacy of chemotherapy, which makes necessary to search for sensitizers of cancer cells to DOX and effective protectors against DOX-induced side-effects [17]. It has been suggested that concomitant administration of DOX with proper antioxidant could attenuate undesired side-effects of chemotherapy, mainly cardiotoxicity, with simultaneous maintaining of drug cytotoxicity towards tumour cells. In addition, an approach has emerged to increase the effectiveness of chemotherapy through introduction of changes in the ratio of membrane components and fluidity, which could allow to overcome the drug resistance and increase sensitivity of cancer cells to anticancer drugs. This therapeutic option has been suggested as the most promising strategy based on modulation of the membrane lipids [18].

Nitroxides have been shown to be potent catalytically acting antioxidants and suggested as suitable to be administered with anticancer drugs during chemotherapy [19,20,21]. For instance, pyrroline nitroxide (Pirolin) has been shown to protect against DOX-induced LPO in rat hearts [22] and against oxidative damage evoked by DOX in the rat brain [21]. Besides its antioxidative action piperidine nitroxide Tempol has been shown to sensitize cancer cells to DOX [23]. One of the group of nitroxides with the potential to gain further interest are doxyl derivatives of stearic acid, of which chains normally occur in cell membranes. Their molecules are spin-labelled at different carbon atoms in lipid acyl chain with oxazolidine ring bearing nitroxyl group. Location of the nitroxyl group along the lipid chain determines the depth on which this group can react and establishes both probing properties and potential effects on the cell membrane. So far, doxyl nitroxides have been widely applied as spin probes used to examine the state of the cell plasma membrane [24], especially its microfluidity, but also fluidity of micellar and liposomal membranes [25]. However, the influence of doxyl nitroxides on plasma membranes and whole cells has not been widely explored. Furthermore, their possible potential to affect multiple cellular changes induced by DOX has not yet been investigated.

**Figure 1:**
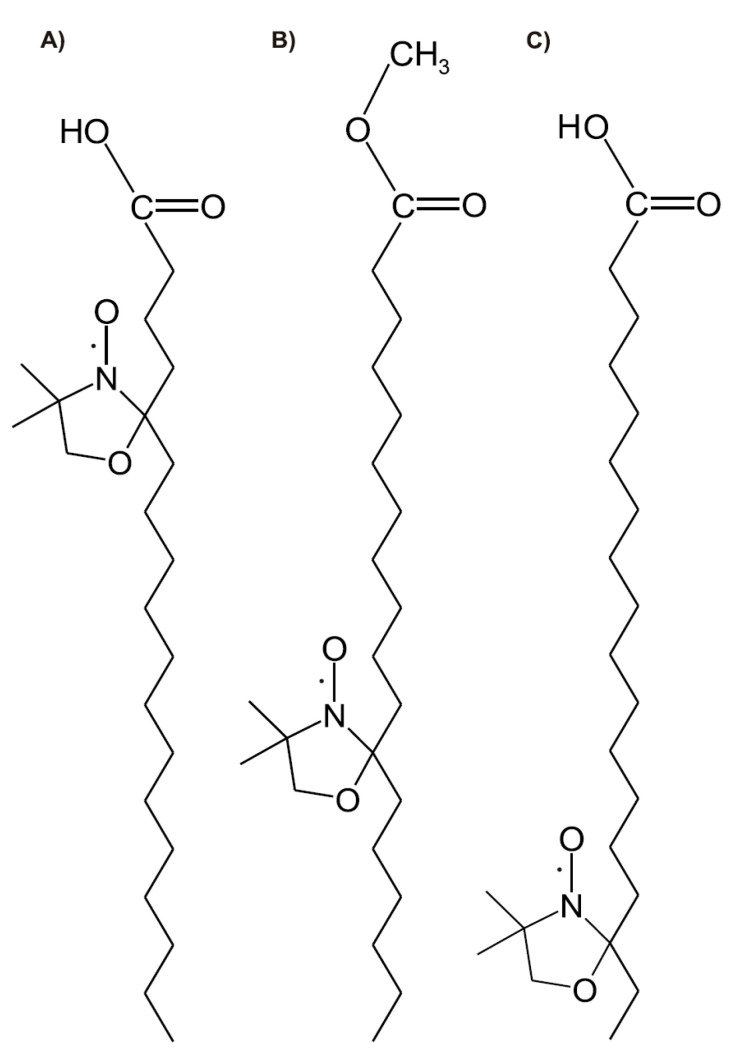
The structure of three derivatives of stearic acid applied in this study spin-labelled using an oxazolidine ring bearing a nitroxyl group: (**A**) 5-doxyl-stearic acid (5-DS), (**B**) 12-doxyl-stearic acid methyl ester (Met-12-DS), and (**C**) 16-doxyl-stearic acid (16-DS).

In this study, we examined the performance of doxyl stearate nitroxides (DSs): 5-doxyl-stearic acid (5-DS; Figure 1A), methyl 12-doxyl-stearate (Met-12-DS; Figure 1B), and 16-doxyl-stearic acid (16-DS; Figure 1C), which possess a nitroxyl group locating at the different depth in the plasma membrane, applied as a single compound and in combination with DOX, in order to evaluate their toxicity towards cells and ability to induce cell apoptosis, changes in plasma membrane fluidity, and to modify toxicity and membrane pro-oxidative activities of DOX, such as perturbations of cell membrane structure, induction of LPO, and cell death. We also evaluated the rates of reduction of DSs added to cells alone and in the presence of DOX. DSs were applied in a wide range of concentrations (1–2000 μM), which covers not only concentration typically used for spin-labelling of erythrocytes, fibroblasts, or other cells in electron paramagnetic resonance (EPR) measurements (100 μM) but also lower (1–50 μM) and higher (200, 500, 1000, and 2000 μM) ones.

A-Tocopherol, the main component of vitamin E, was used as a reference compound. Its hydrophobic molecule has a long saturated phytyl chain [26] and localizes in cell membranes similarly to phospholipids and possibly doxyl nitroxides [27]. For many years, α-Tocopherol has been regarded as a main exogenous antioxidant in the human body. Thus, it has been proposed as a supportive compound, which might be included in chemotherapy, particularly together with anticancer drugs, producing ROS and evoking oxidative stress, such as DOX. Moreover, its synthetic analogues have been found to display anticancer effects alone or to enhance the effects of anticancer drugs, including DOX [28].

The investigations were performed on immortalised fibroblasts (B14 cell line), used as a model for cells with neoplastic phenotype. Earlier, B14 cells have been shown to possess a plasma membrane susceptible to DOX-induced changes [4].

## 2. Results

### 2.1. Cell Viability

#### 2.1.1. The Effects of the Investigated Compounds Applied Alone

**Figure 2:**
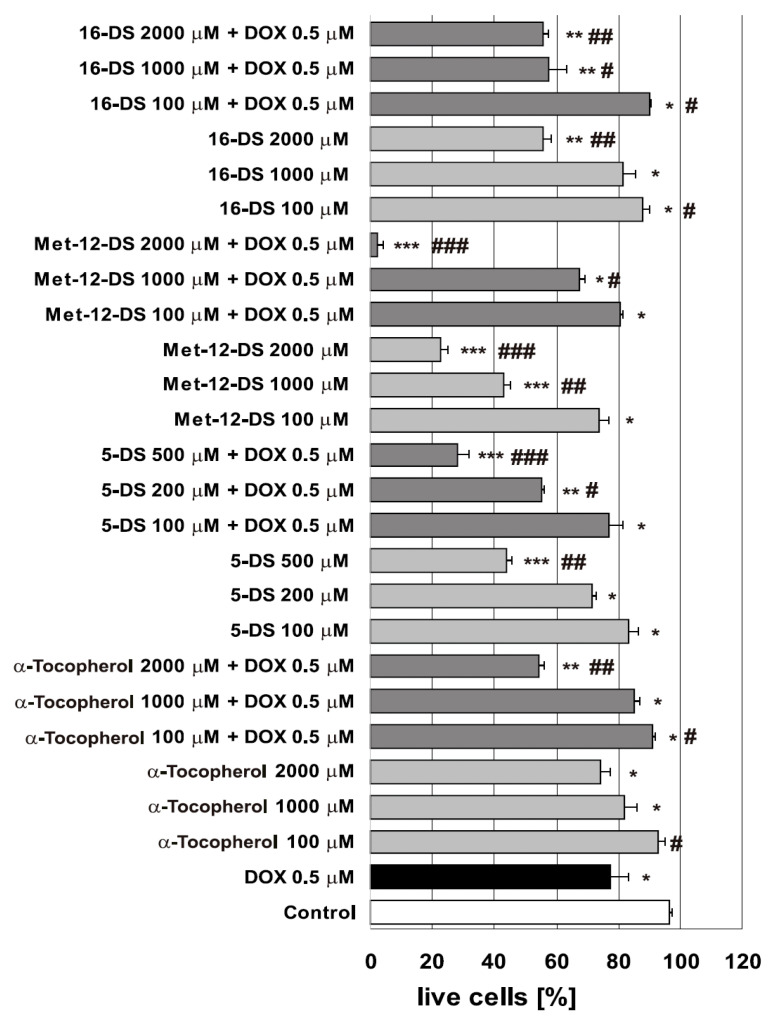
Cell viability of B14 fibroblasts determined by the trypan blue exclusion test. The cells were incubated for 3 h with α-Tocopherol and doxyl stearate nitroxides: 5-DS, Met-12-DS, and 16-DS or for 2 h with doxorubicin (DOX). In combined treatment cells before incubation with DOX were pretreated for 1 h with α-Tocopherol or with any of the doxyl stearate nitroxides. At the end of the incubation (0 h time point) with the investigated compounds, cells were stained with trypan blue (0.2% final concentration of the dye). All cells and blue stained dead cells were counted with an Olympus IX70 microscope (Tokyo, Japan), and the fraction of live cells was calculated as a percentage of all cells (100%). The data are shown as a mean ± SD of three independent experiments, each in at least three repeats.* *p* < 0.05 vs. control; ** *p* < 0.005 vs. control; *** *p* < 0.001 vs. control; ^#^ *p* < 0.05 vs. DOX; ^##^ *p* < 0.005 vs. DOX; ^###^ *p* < 0.001 vs. DOX.

Doxyl stearate nitroxides, applied alone, showed a concentration-dependent toxicity towards B14 cells. High concentrations of 5-DS (500 μM) and Met-12-DS (1000 and 2000 μM) displayed the highest toxicity (Figure 2). Whereas 100 μM of any of the DSs and 1000 μM of 16-DS decreased cell viability by about 10–20%, an effect comparable with that of 0.5 μM DOX, 500 μM 5-DS, and 1000 μM Met-12-DS displayed considerably higher cytotoxicity leading to about a 55% decrease in the population of live cells. The 1000 μM concentration of 16-DS showed comparable effect to that of α-Tocopherol. At the highest concentration (2000 μM), 16-DS was considerably less cytotoxic than Met-12-DS, while α-Tocopherol was the least toxic compound. Both 16-DS and α-Tocopherol were considerably less toxic than 5-DS and Met-12-DS.

#### 2.1.2. The Effects of the Investigated Compounds Applied in Combination With 0.5 μM DOX

Pretreatment of cells with the investigated compounds before their incubation with DOX, differently affected DOX cytotoxicity. Only α-Tocopherol and 16-DS at 100 μM concentration acted protectively and caused a moderate (about 13%) increase in the fraction of live cells. Neither 5-DS nor Met-12-DS at this concentration displayed any effect. Higher concentrations of nitroxides and α-Tocopherol enhanced DOX cytotoxicity to a different degree. The greatest effects were observed for 200 and 500 μM 5-DS (45 and 70% reduction in fraction of live cells, respectively, compared to 20% reduction caused by 0.5 μM DOX). Met-12-DS was also considerably toxic, and at 2000 μM concentration, it eliminated more than 95% of viable cells. Significant enhancement of DOX toxicity was also evident in cells preincubated with 1000 and 2000 μM 16-DS and 2000 μM α-Tocopherol, which caused a loss of about 45% of the live cell population (Figure 2).

### 2.2. Changes in Cell Morphology and Induction of Cell Death

#### 2.2.1. The Effects of Doxyl Stearate Nitroxides and α-Tocopherol

**Figure 3:**
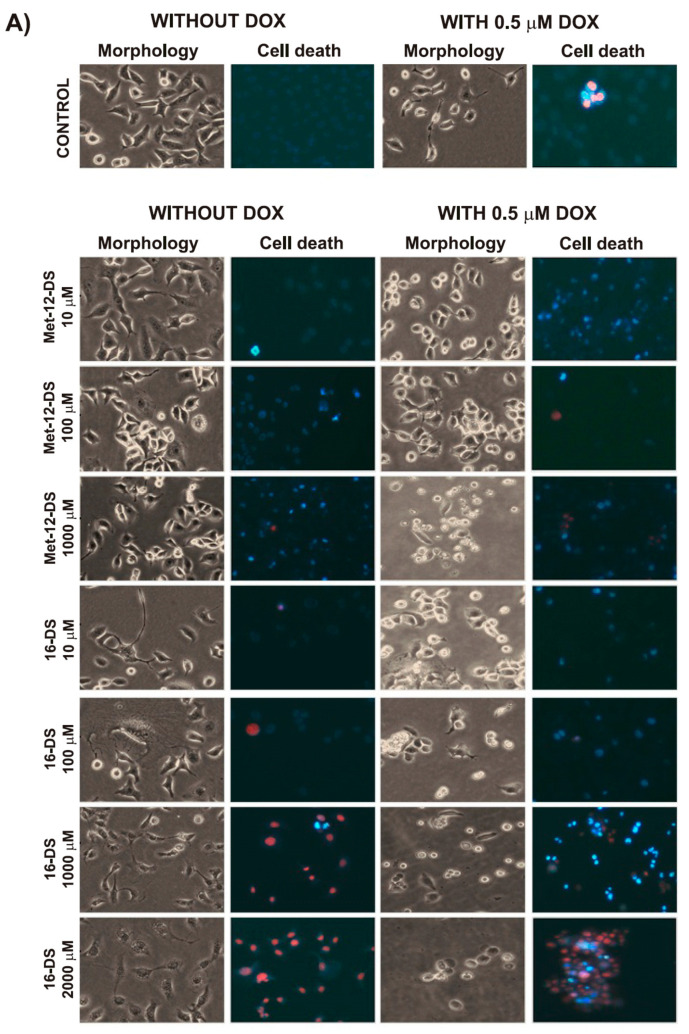

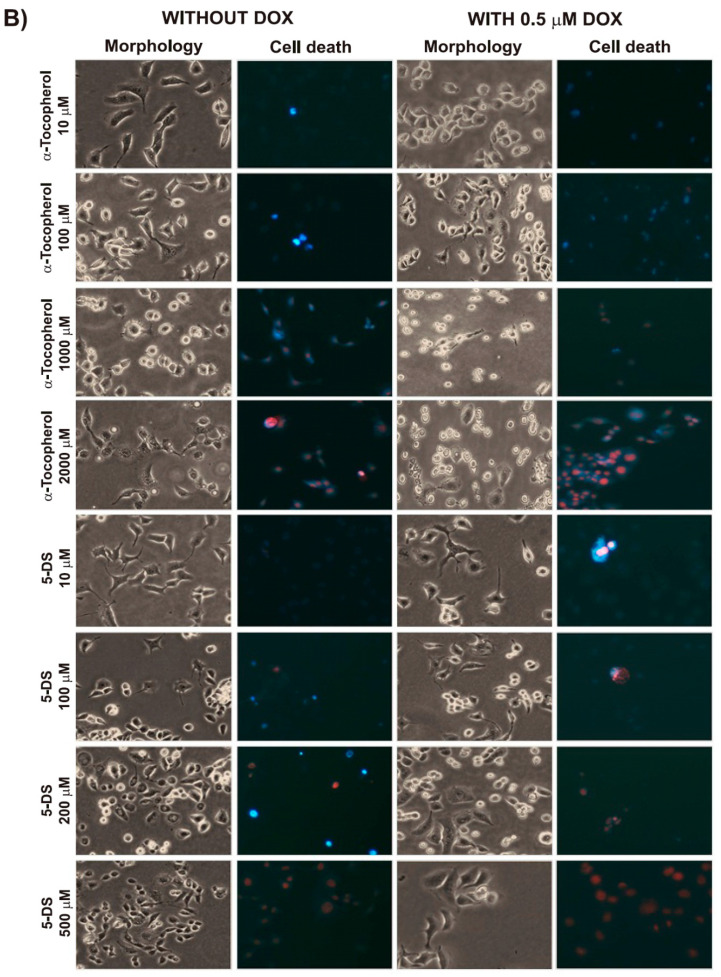
Morphology and cell death of B14 cells at 24 h after cell treatment with the investigated compounds administered alone (0.5 μM doxorubicin (DOX), α-Tocopherol, and doxyl stearate nitroxides: 5-DS, Met-12-DS, and 16-DS) or in combination: 0.5 μM DOX with α-Tocopherol or with the nitroxides. Cell death was visualized using fluorescent double staining with propidium iodide (PI) and Hoechst 33258. Cell monolayers were analysed using an optical microscopy (morphology) or with the use of an inverted fluorescence microscopy (cell death) (Olympus IX70, Tokyo, Japan) under magnification 150×. Cells were classified as viable (blue fluorescent), early apoptotic (bright blue fluorescent), late apoptotic (blue-violet fluorescent), and necrotic (red fluorescent cells). (**A**) Control cells, DOX alone, Met-12-DS or 16-DS-treated cells; (**B**) α-Tocopherol, 5-DS.

**Figure 4:**
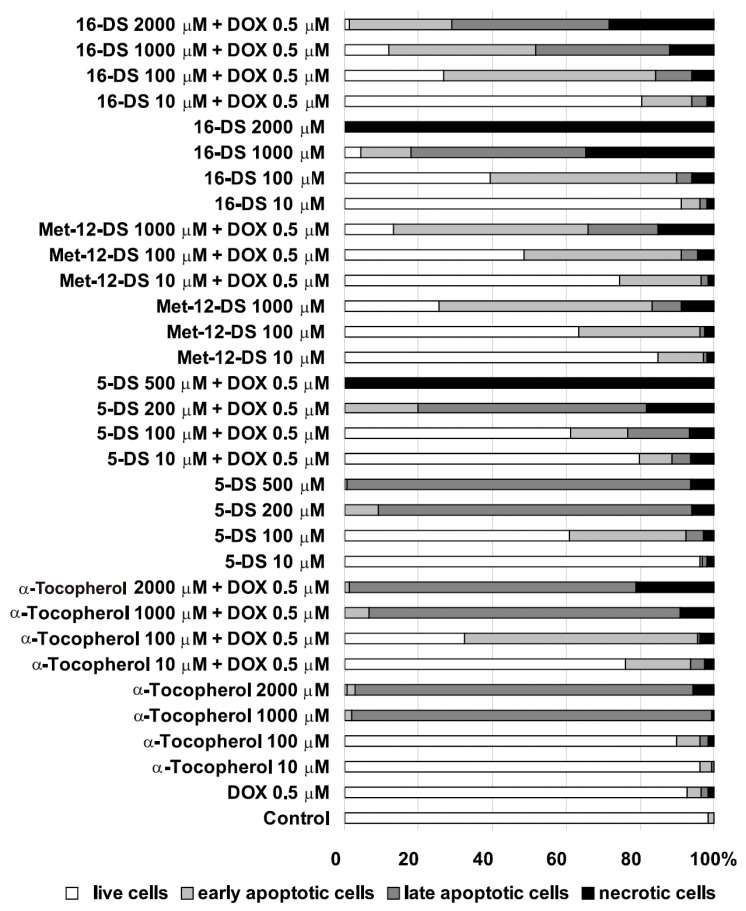
Percentages of live, early apoptotic, late apoptotic, and necrotic cells at 24 h after B14 cell treatment with α-Tocopherol; doxyl stearate nitroxides: 5-DS, Met-12-DS, and 16-DS, administered alone or in combination with 0.5 μM doxorubicin (DOX). Changes in cell morphology associated with apoptosis/necrosis were determined based on fluorescent double staining with propidium iodide (PI) and Hoechst 33258 (Section 2.2 and Section 4.2.3, Figure 3). The percentages of particular cell fractions were calculated in relation to all cells (100%).

Our study showed that 5-DS, Met-12-DS, and 16-DS, as well as a reference compound α-Tocopherol, can trigger apoptosis, which intensity increased with an increase in compound concentrations. The level of spontaneous cell death observed in control cells was negligible and did not exceed 2% (Figure 3A and Figure 4). A concentration of 10 μM of 5-DS, 16-DS, and α-Tocopherol induced minor changes (the fraction of apoptotic and necrotic cells did not exceed 10%). A significant, progressive increase in the percentage of apoptotic cells was observed after treatment with high concentrations of nitroxides. The highest intensity of apoptosis was found in cells incubated with 5-DS. Low and intermediate concentrations (10 and 100 μM) of α-Tocopherol had a small influence on cell viability. Some features typical for early apoptosis, such as cell shrinkage, however, were visible in cells incubated with 10 μM concentration of α-Tocopherol and to a greater extent in cells treated with its 100 μM concentration (Figure 3B and Figure 4). A high concentration of α-Tocopherol (1000 μM) caused cells to lose their normal morphological features and enter the apoptosis pathway. Besides shrunken apoptotic cells, enlarged necrotic cells were also present, however, most of the cells were in late apoptosis (Figure 3B and Figure 4). Incubation with 2000 μM α-Tocopherol considerably intensified cell death processes (Figure 3B and Figure 4).

In the case of 5-DS alone, at the 10 μM concentration, it induced small changes in the cell morphology, and only about 4% of the cells were classified as early apoptotic, late apoptotic, or necrotic (Figure 3B and Figure 4). The effects were more visible after treatment with 100 μM concentration of the nitroxide, which induced changes typical for apoptosis—altered morphology and cell shrinkage. About 37% of the cells were in apoptotic cell death (Figure 3B and Figure 4). Concentrations of 200 and 500 μM of 5-DS augmented cell shrinkage and increased intensity of apoptosis, which in its late phase, was found in most of the cells (Figure 3B and Figure 4).

The lowest concentration of Met-12-DS (10 μM) induced an early apoptosis in about 12% of the cells. Changes in the cell shape, cell shrinkage, and enhancement of early apoptosis (about 35%) were detected for 100 μM Met-12-DS and, particularly, for its 1000 μM concentration, which evoked a large increase in the incidence of early apoptosis. About 8% of the cells were in the late stage of apoptosis or were dead (about 9%) (Figure 3A and Figure 4).

In the case of 16-DS alone, already at the lowest concentration (10 μM), it induced changes in cell morphology and an early apoptosis in about 5% of the cells. In some cells, late apoptosis and necrosis were detected. The changes were most evident for the high concentrations of 16-DS: at 100 μM necrosis was also observed and 1000 μM nitroxide induced all types of cell death: an early and late apoptosis and necrosis in the considerable percent of cells. At 2000 μM concentration of 16-DS, all cells were necrotic, but also polyploidisation and formation of two nuclei in some of the cells were noted (Figure 3A and Figure 4).

#### 2.2.2. The Effects of the Investigated Compounds Applied in Combination With 0.5 μM DOX

After 2 h treatment with 0.5 μM DOX followed by a 24 h post-incubation in a fresh medium, loss of normal morphological features of B14 cells, changes in the shape of the cell, and cell shrinkage were observed. In DOX-treated cells the total number of cells undergoing cell death did not exceed 8%; however, both an early and late apoptosis or necrosis evoked by the drug were detected (Figure 3A and Figure 4).

Combined treatment of cells with 10 μM α-Tocopherol and 0.5 μM DOX changed the shape and evoked cell shrinkage of almost all cells. More than 17% of the cells were classified as early apoptotic and only 6% as late apoptotic or necrotic (Figure 3B and Figure 4). The observed changes were enhanced after treatment with 100 μM α-Tocopherol (Figure 3B and Figure 4). Combination of 1000 μM α-Tocopherol and 0.5 μM DOX was highly toxic to B14 cells, and the majority of cells were in late apoptosis (Figure 3B and Figure 4). The most harmful changes were induced by the highest concentration of α-Tocopherol—late apoptosis was detected in more than 75% of cells and about 22% of cells appeared as enlarged necrotic cells (Figure 3B and Figure 4).

A concentration of 10 μM of 5-DS in combination with 0.5 μM DOX induced the formation of polyploid cells with two or more nuclei and caused cell shrinkage. Early apoptotic (9%), late apoptotic (5%), and necrotic (6%) cells were present (Figure 3B and Figure 4). A concentration of 100 μM of 5-DS increased the number of shrunken cells. About 15% of cells were in an early or late apoptosis or in necrosis (6%). Late apoptotic cells were prevalent after cell incubation with 200 μM 5-DS, and in addition, higher percent of enlarged cells could be observed than in cells treated with lower concentrations. Nearly 20% of cells in necrosis were also detected. The highest (500 μM) concentration of 5-DS in combination with DOX caused necrosis in all cells (Figure 3B and Figure 4).

A concentration of 10 μM of Met-12-DS combined with DOX induced changes typical for early apoptosis, which were observed in 22% of the cells. A concentration of 100 μM of the nitroxide enhanced this effect, and part of the cells proceeded to late apoptosis and necrosis (Figure 3A and Figure 4). A combination of 1000 μM Met-12-DS and DOX induced apoptosis of most of the cells. Early apoptotic cells accounted for more than 50%, but late apoptotic cells with apoptotic bodies and necrotic cells were also visible (Figure 3A and Figure 4).

More cells incubated with 10 μM 16-DS in combination with 0.5 μM DOX showed features of apoptosis (changed shape, cell shrinkage) than cells incubated with 10 μM 16-DS alone. Morphological changes were altered by higher concentrations of 16-DS combined with DOX in comparison to this nitroxide alone (Figure 3A). Interestingly, that preincubation of DOX-treated cells with high concentration of 16-DS (1000 μM) increased fraction of early apoptotic cells and decreased fractions of late apoptotic and necrotic cells (Figure 3A and Figure 4). A concentration of 2000 μM of 16-DS combined with DOX delayed the induction of cell death in comparison with nitroxide alone: about 28% of the cells were in an early apoptosis, more than 40% in late apoptosis, and about 28% were dead (Figure 3A and Figure 4). In comparison with DOX alone, the combination of 16-DS with DOX caused an enhancement of apoptosis or necrosis, depending on the concentration of the nitroxide (Figure 3A and Figure 4).

### 2.3. Membrane Fluidity

#### 2.3.1. The Effect of Doxyl Stearate Nitroxides and α-Tocopherol

**Figure 5:**
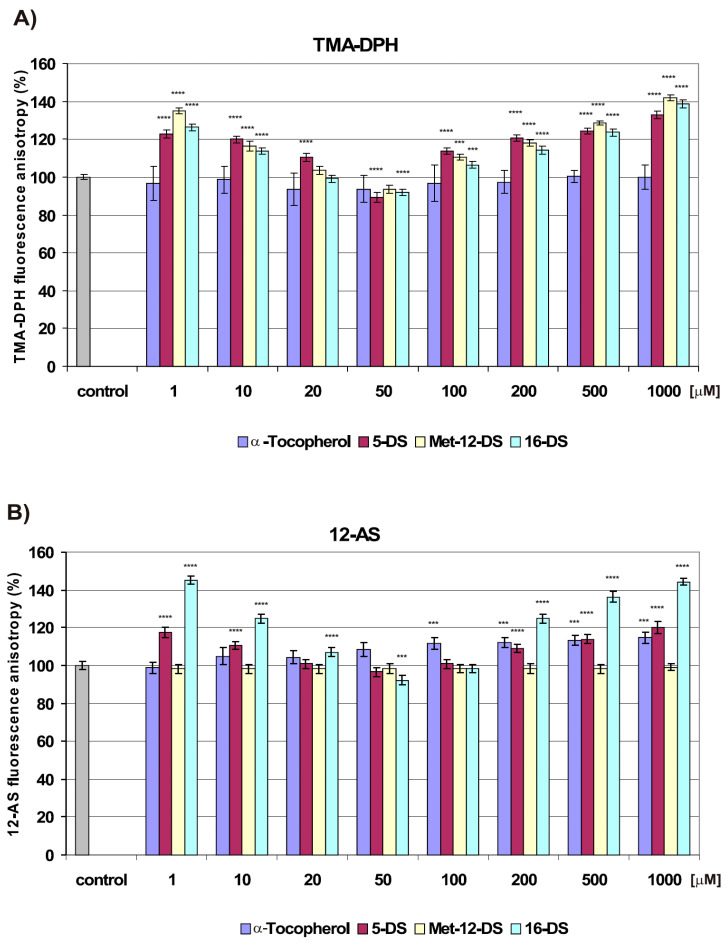

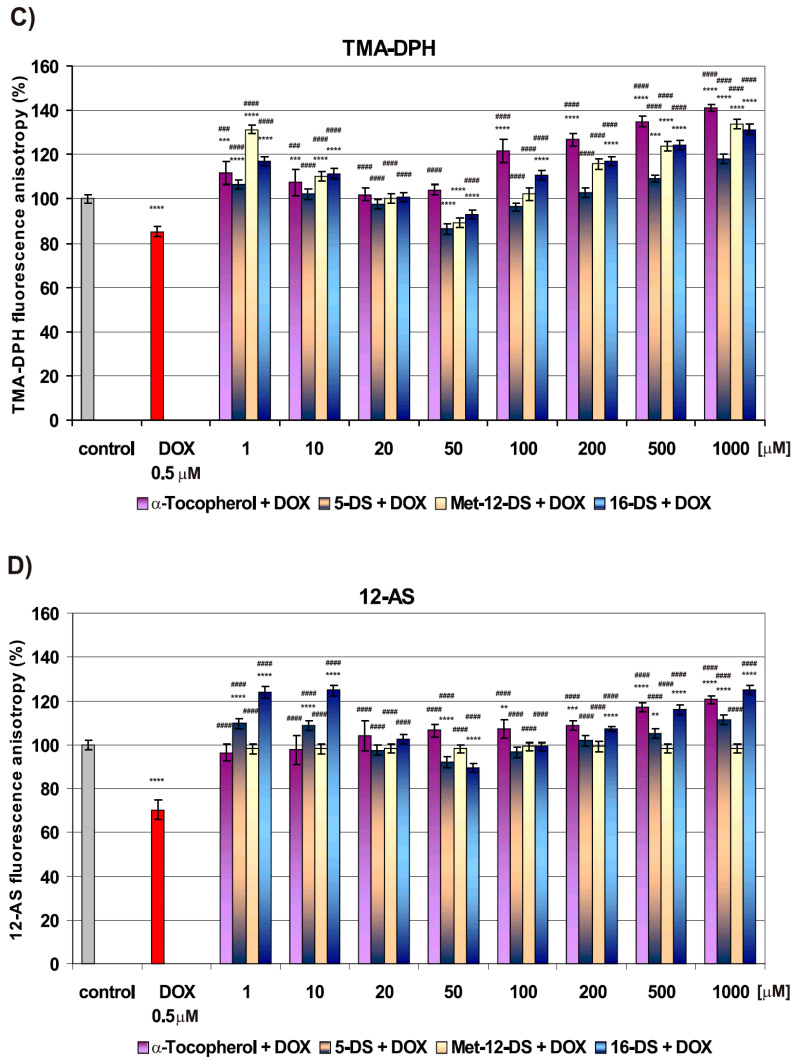
The influence of doxyl stearate nitroxides: 5-DS, Met-12-DS, and 16-DS and α-Tocopherol administered alone (**A**,**B**) or in combination with 0.5 μM doxorubicin (DOX) (**C**,**D**) on fluidity of the plasma membrane of B14 cells measured fluorimetrically using 1-(4-Trimethylammoniumphenyl)-6-Phenyl-1,3,5-Hexatriene *p*-Toluenesulfonate (TMA-DPH) (**A**,**C**) and 12-AS (**B**,**D**) fluorescent probes (Section 4.1.1 and Section 4.2.4). The data are shown as a mean ± SD. ** *p* < 0.01 vs. control; *** *p* < 0.001 vs. control; **** *p* < 0.0001 vs. control; ^###^ *p* < 0.001 vs. DOX; ^####^ *p* < 0.0001 vs. DOX.

Doxyl stearate nitroxides basically caused a concentration-dependent decrease in fluidity of lipid bilayer (Figure 5A,B). Similar biphasic mode of changes was observed after treatment with any of the investigated DSs—an initial decrease in plasma membrane fluidity, evoked by incubation with low nitroxide concentrations (0–20 μM), followed by an increase at 50 μM, and another decrease triggered by higher concentrations (100–1000 μM).

No changes in fluidity of the surface region of lipid bilayer at 20 μM Met-12-DS and 20 μM 16-DS (Figure 5A) and in the hydrophobic core at 20–100 μM 5-DS (Figure 5B) were observed. Changes induced by Met-12-DS were not measurable with 12-AS fluorescent probe due to fluorescence quenching by Met-12-DS (Figure 5B). Interestingly, that DSs at a concentration of 50 μM induced a minor (about 10%) increase in the fluidity of the surface part of the plasma membrane (Figure 5A). Fluidisation of the membrane hydrophobic core was observed after incubation with 50 μM 16-DS (Figure 5B). A further gradual increase in the nitroxide concentration to 1000 μM caused a progressive 10–45% decrease in fluidity of both membrane regions. To summarize, all three DSs had a similar impact on the fluidity of the surface part of the membrane, but 16-DS induced considerably greater changes than 5-DS in its hydrophobic region (Figure 5A,B).

In contrast to DSs, α-Tocopherol influenced only on the deeper, hydrophobic part of the membrane lipid bilayer and, at high concentrations (100–1000 μM), caused a moderate (15% on average) decrease in membrane fluidity (Figure 5B).

#### 2.3.2. The Effect of Doxyl Stearate Nitroxides and α-Tocopherol on Changes in Fluidity of Plasma Membrane Induced by DOX

Doxorubicin at 0.5 μM concentration induced fluidisation of the cell membrane, which is in compliance with our earlier study [4], and this effect was more visible in the hydrophobic part of the membrane (Figure 5C,D).

Pretreatment of cells with any of the DSs significantly modified changes in the structure of the plasma membrane caused by DOX. At some of the examined concentrations, DSs attenuated drug effects or caused opposite changes—plasma membrane rigidification. Membrane fluidity was restored to the level of control by 5-DS at 10–20 and 100–200 μM concentrations for measurements with 1-(4-Trimethylammoniumphenyl)-6-Phenyl-1,3,5-Hexatriene *p*-Toluenesulfonate (TMA-DPH) (Section 4.1.1 and Section 4.2.4), and at 20 and 100–200 μM concentrations for measurements with 12-AS (Figure 5C,D). Similar concentrations (20 and 100 μM) of Met-12-DS also reversed changes evoked by DOX and normalised fluidity of the membrane surface region to the fluidity of control cells. Reversal of membrane fluidity in cells pretreated with 16-DS was detected for fewer concentrations than in the case of 5-DS, i.e., for 20 μM 16-DS in the surface region of the membrane (Figure 5C) and for 20 and 100 μM 16-DS in the hydrophobic part of the membrane (Figure 5D).

None of the DSs enhanced DOX effect on membrane fluidity. Comparison of changes in membrane fluidity induced by doxyl nitroxides alone and by their combination with DOX suggests the dominating effect of nitroxides, expressed in the same biphasic mode of induced changes in membrane fluidity as in experiments with DSs alone. Due to the membrane fluidisation effect of DOX, which counteracts the membrane rigidification effect of doxyl nitroxides, DSs applied alone caused a larger decrease in membrane fluidity than their combination with DOX.

Low 1 μM and high (500 and 1000 μM) concentrations of 5-DS in combination with 0.5 μM DOX caused rigidification of both membrane regions; the effect being opposite to that of DOX (Figure 5C,D). In combination with DOX, Met-12-DS and 16-DS had a more pronounced effect than 5-DS and caused stronger rigidification of the plasma membrane even at low concentrations (1 and 10 μM). Changes induced by Met-12-DS concern only the surface region of the membrane, as changes in fluidity of its hydrophobic part were not measurable with 12-AS fluorescent probe due to fluorescence quenching by Met-12-DS.

A-Tocopherol at 20–50 μM concentrations fully reversed changes in membrane fluidity in the surface region of the plasma membrane induced by DOX (Figure 5C). At the same time, 1 and 10 μM concentrations of α-Tocopherol were sufficient to reverse changes in fluidity of the hydrophobic membrane region (Figure 5D). Higher concentrations of α-Tocopherol caused rigidification of the membrane both in comparison to control and DOX, and this effect was the most distinct for its highest concentrations (500 and 1000 μM) (Figure 5C,D). Compared with DSs, α-Tocopherol in combination with DOX induced stronger rigidification of the surface part of the plasma membrane, expressed as a 20–40% decrease in membrane fluidity compared with control and in a broader range of concentrations (100–1000 μM) (Figure 5C).

### 2.4. Lipid Peroxidation

#### 2.4.1. The Effect of Doxyl Stearate Nitroxides and α-Tocopherol

**Figure 6:**
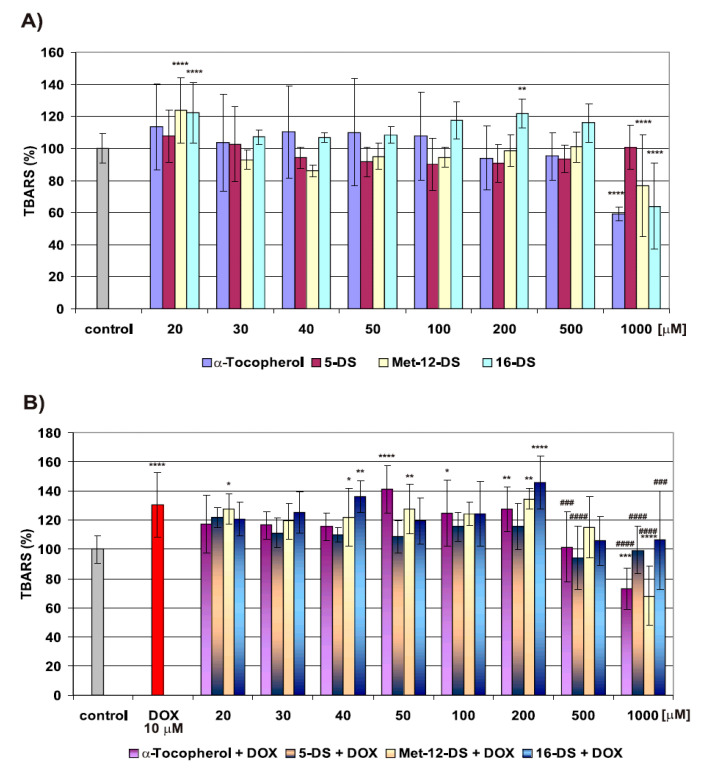
(**A**) The level of lipid peroxidation estimated on the basis of measurements of the amount of thiobarbituric acid-reactive substances (TBARS) in the plasma membrane of B14 cells treated with doxyl stearate nitroxides (5-DS, Met-12-DS, and 16-DS) and α-Tocopherol. The data are shown as a mean ± SD of three independent experiments, at least in three repeats each. ** *p* < 0.02 vs. control; **** *p* < 0.0001 vs. control. (**B**) The effect of α-Tocopherol and doxyl stearate nitroxides 5-DS, Met-12-DS, and 16-DS on lipid peroxidation induced in the plasma membrane of B14 cells by 10 μM doxorubicin (DOX). The data are shown as a mean ± SD of three independent experiments, at least in three repeats each. * *p* < 0.05 vs. control; ** *p* < 0.01 vs. control; *** *p* < 0.001 vs. control; **** *p* < 0.0001 vs. control; ^###^ *p* < 0.001 vs. DOX; ^####^ *p* < 0.0001 vs. DOX.

For the estimation of LPO (assayed as the amounts of thiobarbituric acid-reactive substances, TBARS) induced in B14 cell plasma membrane, DSs and α-Tocopherol were used in the 20–1000 μM concentration range (Figure 6A,B). A concentration of 2000 μM was not included due to its high cytotoxicity towards B14 cells (Figure 2).

We did not find any influence of 5-DS on LPO. Met-12-DS and 16-DS, however, displayed antioxidant properties and, similar to α-Tocopherol at the highest concentration (1000 μM), inhibited the formation of TBARS and decreased their amount by about 25% (Met-12-DS) and 35% (16-DS) in relation to control (Figure 6A). Both nitroxides at low concentration (20 μM) and 16-DS additionally at 200 μM concentration caused about a 20% increase in TBARS.

#### 2.4.2. The Effect of Doxyl Stearate Nitroxides and α-Tocopherol on LPO Induced by DOX

In our earlier study, we have found that DOX, during 2 h incubations with B14 cells, displayed different effects on plasma membrane fluidity, depending on concentration. The drug affected the structure of the cell membrane to the highest extent at higher concentrations—10 and 20 μM [4]. In another study, we reported insignificant LPO in the plasma membrane of B14 cells treated with a LC50 concentration of DOX and a significant increase in LPO after treatment of cells with 10 μM DOX [29]. Based on these results, we chose 10 μM DOX for the current experiments.

In the present study, we have found that 10 μM DOX increased LPO amount by 30% on average (Figure 6B). Neither DSs nor α-Tocopherol in the concentration range of 20–200 μM influenced LPO induced by DOX (Figure 6B). In combination with 10 μM DOX, α-Tocopherol at 500 μM concentration decreased the TBARS amount to the level of control and strongly inhibited LPO at a higher concentration. About a 25% decrease in TBARS amount in relation to control was observed in B14 cells preincubated with 1000 μM α-Tocopherol (Figure 6B). Comparable level of LPO inhibition was also observed in cells pretreated with the same concentration of Met-12-DS. High concentrations of 5-DS (500 and 1000 μM), also decreased to the level of control LPO induced by DOX (Figure 6B). In the same range of concentrations 16-DS displayed a weaker protective effect (Figure 6B).

### 2.5. Reduction of Doxyl Stearate Nitroxides

EPR technique was employed to investigate the stability of the used DSs in time in solution and the possibility of their interactions with DOX, the rate of reduction of the DSs in B14 cells, and to check the influence of DOX on bioreduction of the DSs.

Both for solutions of nitroxides applied alone and for solutions of DSs mixed with DOX (all compounds at 80 μM final concentration) no significant changes in the EPR signal intensity were detected within 24 h (data not shown).

For the DSs added to cells alone (all nitroxides at 80 μM final concentration) or together with DOX (1 μM DOX, 80 μM DS) a consecutive lowering of the EPR signal intensity related to the reduction of the DSs in cells was observed in time according to a linear function (Section 4.2.6, Equation (2)).

**Figure 7:**
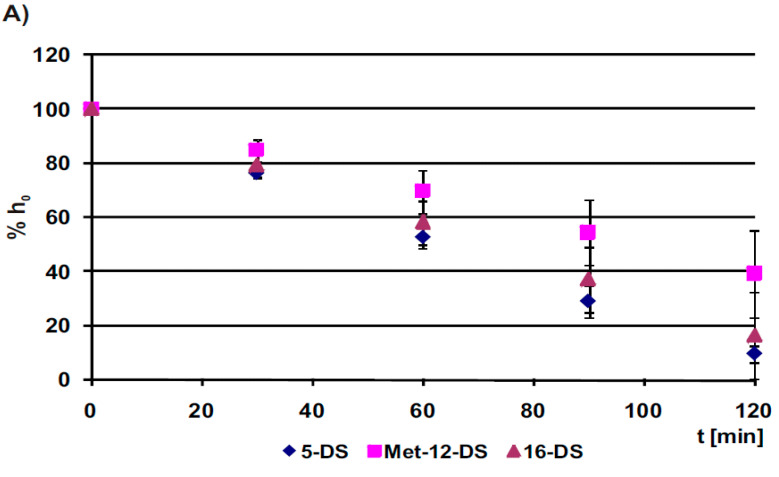

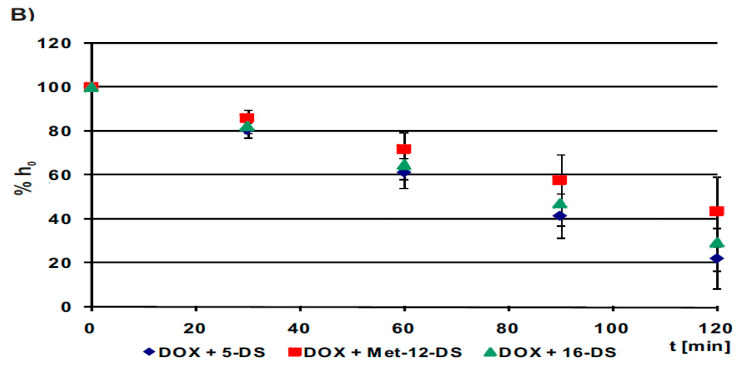
Kinetics of the reduction by B14 fibroblast cells of the doxyl stearate nitroxides (DSs): 5-DS, Met-12-DS, and 16-DS. The data are shown as a percentage of the height of the middle-field line (h_0_), calculated based on the spectra of the DSs obtained from EPR measurements (Section 4.2.6, Equation (2)). All the data are shown as a mean ± SD of at least three independent experiments. (**A**) Reduction by B14 cells of 5-DS, Met-12-DS, and 16-DS applied alone at 80 μM final concentration. (**B**) Reduction by B14 cells of 5-DS, Met-12-DS, and 16-DS applied at 80 μM final concentration together with 1 μM doxorubicin (DOX).

Among the investigated nitroxides applied alone, 5-DS was reduced in B14 cells at the fastest rate (k), while Met-12-DS at the slowest (Figure 7A, Table 1A). EPR measurements revealed that 5-DS had the time of half-reduction (*t*_½_) of approximately 63 min, while for Met-12-DS half-reduction time was calculated to be about 1.6 times longer, and this difference was statistically significant (Table 1B).

On the other hand, for all the DSs added to cells together with DOX, a noticeable slowing of reduction was observed (Figure 7B, Table 1B), which resulted in elongation of half-reduction times. In combination with 1 μM DOX, all of the DSs times of half-reduction were longer for about 20 min than for nitroxides added to cells alone, and the greatest influence of DOX was detected for 5-DS (Table 1A,B). However, none of the changes induced by DOX were statistically significant in relation to nitroxides alone (Table 1A,B). Simultaneously, the general pattern of the speed of nitroxide reduction in the presence of DOX was saved, i.e., 5-DS was reduced in cells the quickest, while Met-12-DS the slowest (Figure 7B, Table 1B).

## 3. Discussion

A-Tocopherol is regarded as one of the main hydrophobic antioxidants, which locates in membranes mostly at the sites rich in polyunsaturated acid residues, where it can play its role of a chain-breaking antioxidant [30]. However, in our study, only the highest concentration (1000 μM) of α-Tocopherol, applied alone, acted protectively and significantly reduced the level of LPO in B14 cells. Similarly, α-Tocopherol acted as an antioxidant and inhibited LPO induced by DOX only at the highest concentrations (500 and 1000 μM).

Partial protection provided by α-Tocopherol is in accordance with other studies. It has been shown that, although α-Tocopherol incorporates into cardiac lipid membranes and protects from oxidative stress, it does not shield against an impairment of mitochondrial function and histopathology of cardiomyocytes [31]. Although α-Tocopherol for many years has been regarded as one of the main hydrophobic antioxidants, its protective properties against cardiotoxicity induced by DOX have not been confirmed [32,33]. In our study, α-Tocopherol, in comparison with DSs, displayed a weaker influence on membrane fluidity and induced its decrease only in the hydrophobic core.

Tiurin et al. [34] reported that the rate of flip-flop of α-Tocopherol molecules is very slow and its amounts in outer and inner monolayers of lipid bilayer can remain unchanged for many hours [34]. Additionally, the study of Urano and co-workers [35] has shown that α-Tocopherol has a membrane stabilising effect, via formation of the hydrogen bond between its hydroxyl group and the carbonyl group of a fatty acid ester of a phospholipid in the bilayer of liposomes. Taken together, these effects can explain the lowest toxicity towards B14 cells among all the investigated compounds.

Some reports exist, which indicate that α-Tocopherol is not able to induce apoptosis [36]. The present study, however, demonstrates that α-Tocopherol can induce apoptosis in a broad range of concentrations (10–2000 μM). Most of the cells treated with its higher concentrations, independently on whether it was administered alone or in combination with DOX, appeared to undergo late apoptosis. Moreover, α-Tocopherol at a low concentration (10 μM) enhanced the toxic effect of DOX and increased the fraction of apoptotic cells. These results support other reports showing that synthetic analogues of α-Tocopherol can induce anticancer effects alone or enhance the anticancer effects of DOX [28].

The cytotoxic properties of DSs have been assessed earlier in different types of cells. For instance, Ankel and co-workers [37] reported lack of cytotoxicity of DSs towards Chinese hamster ovary (CHO) cells. Our results of the cytotoxic effect of the investigated nitroxides at 100 μM concentration are in partial contradiction to the results obtained earlier, which showed that at this concentration only 5-DS had an inhibitory effect on survival of CHO cells, while 12-DS and 16-DS did not show any effect [37]. We showed the cytotoxic effect of 100 μM concentration of the DSs, which is commonly used for the spin-labelling of cells in EPR measurements. This discrepancy could be related to the different type of cells used in both studies, which might determine their different susceptibility to the investigated nitroxides. We examined concentrations of up to 2000 μM for α-Tocopherol, Met-12-DS, and 16-DS and showed substantial toxicity of high concentrations of the nitroxides towards B14 cells. Furthermore, the doxyl nitroxides 5-DS, 16-DS, and 12-DS were found to display antibacterial activity and high cytotoxicity towards *Bacillus subtilis* cells, and the position of the doxyl moiety in the acyl chain of the nitroxide was pointed out as the main factor influencing this effect and causing 5-DS to be relatively the most cytotoxic [38]. We have found that 5-DS, in comparison with Met-12-DS and 16-DS, induced more incidences of cell death, but did not significantly influence plasma membrane LPO. This implies that high cytotoxicity of this compound cannot be linked to its direct prooxidant action towards membrane lipids. In our investigation 5-DS has been found to be reduced at the fastest rate among the applied nitroxides, which is a confirmation of previous results obtained from experiments performed on mouse neuroblastoma (NB) cells [39] and RBL-2H3 mast cells [40]. Undifferentiated NB cells reduced 5-DS about 1.6 times faster than 16-DS. These cells have a very high level of non-protein thiol groups, permeation of which from the cytosol to plasma membrane was suggested as the most likely cause of nitroxide reduction [39]. In RBL-2H3 mast cells, the phosphatidylcholine (PC) EPR probe 5PC incorporated into the plasma membrane was reduced faster than 10PC and 12PC [40]. Other reductors, which have been suggested to be involved in the reduction of the nitroxyl group of a lipid spin probe, are free thiol groups of proteins present in the membrane [40]. In comparison to two other DSs investigated in our study, 5-DS, having the nitroxyl group the closest to the surface of the membrane, should be the one that could react easily with surface thiol groups, especially those of the membrane peripheral proteins, which could influence the rate of reduction of 5-DS. Another compound, which has been shown to be present in the membrane bilayer at a relatively high concentration and can act as an effective antioxidant, is ubiquinol [41]. In the model membrane, one of its parts where reduced benzoquinone ring of ubiquinol has been found is a region near the phospholipid glycerol head groups [42]. The presence of a ubiquinol ring near the extracellular surface region of the membrane could also account for an enhancement of the reduction of 5-DS in our study. The action of all the above membrane antioxidants might cause the fastest reduction of 5-DS in comparison to two other DSs. Apparently, the product of the nitroxide reduction, namely its hydroxylamine, might have some account for the effects observed after incubation of cells with the nitroxide. Despite the fastest rate of reduction among the investigated nitroxides, 5-DS, however, caused a decrease in membrane fluidity in a wide range of low and high concentrations.

It is known that alterations in the plasma membrane fluidity induced by different compounds can lead to changes in the function of membrane proteins, cell signalling, and gene expression [43]. Furthermore, nitroxide performance, including its effects on biological membranes, strongly depends on its structure and concentration [29]. This was confirmed in our study for the 50 μM 5-DS, which unlike other concentrations of this nitroxide, increased fluidity in the surface membrane region. Both low and high concentrations of Met-12-DS caused a decrease in the fluidity of the surface part of the membrane bilayer. These changes might contribute to significant toxicity of Met-12-DS; however, this nitroxide at 1000 μM concentration was equally cytotoxic as 500 μM 5-DS. Met-12-DS only at 100 μM concentration induced cell death in part of the cells, comparably to 5-DS.

Above 100 μM concentration, Met-12-DS induced cell death in a smaller percent of cells than 5-DS. Since we did not detect any changes in membrane fluidity (in relation to control) using a 12-AS fluorescent probe, linking the changes in fluidity of the hydrophobic part of the membrane to the toxicity of Met-12-DS is not possible. The complete lack of changes in fluidity, measured with a 12-AS probe, suggests the occurrence of interactions between the paramagnetic EPR probe Met-12-DS and the fluorescent probe 12-AS, which both locate their functional groups at the same depth in the hydrocarbon chain. Since different paramagnetic species can quench fluorescence [44], we can suggest that quenching occurred due to the presence of unpaired electron in the nitroxyl group of Met-12-DS. It has been shown, e.g., that spin-labelled PC quenched the fluorescence of a fluorescent probe diphenylhexatriene (DPH), which is located in the cell membrane deeper than TMA-DPH. Quenching of DPH was found to be static due to contact of spin-labelled phospholipid with the fluorophore [45]. However, the type and strength of interaction(s) between Met-12-DS and 12-AS cannot be explained on the basis of the presented results, and this issue would need further detailed studies.

At 1000 μM concentration 16-DS strongly inhibited LPO, and it seems that observed significant rigidification of cell membrane, induced by this concentration, could also depend on the concentration overload of the membrane caused by 16-DS. Similarly to 5-DS, other concentration dependence of the effects also occurred, since intermediate 16-DS concentration (50 μM) caused membrane fluidisation. Although changes in membrane fluidity caused by 16-DS were similar to those induced by 5-DS, but more pronounced, 16-DS was found to be less cytotoxic than 5-DS and Met-12-DS as determined by the trypan blue exclusion assay. On the other hand, our results demonstrated that cellular changes induced by 16-DS may be prolonged in time, as 24 h later, its 100 and 1000 μM concentrations caused cell death of a higher percentage of cells than Met-12-DS, which conversely, was more cytotoxic in the trypan blue exclusion assay. Some influence on cytotoxicity of 16-DS might have its reduction, slower than in the case of 5-DS, but faster than for Met-12-DS. The rate of nitroxide reduction is probe- and cell-dependent. For instance, the PC derivative 16PC EPR probe has been shown to perform differently than other probes, e.g., 5PC and 12PC, and 16PC was reduced very quickly in NIH-3T3 cells, faster than in other live cells investigated in earlier studies [40]. Moreover, in the work where piperidine nitroxides targeting mitochondrial membrane were studied, it has been suggested that their rates of reduction by ubiquinol were associated with different accessibility of the nitroxyl group to this reductant inside the membrane [46]. For 16PC a slight inclination to incorporate in the membrane segments with less ordered acyl chains of phospholipids has been shown, because their lower order close to the central part of the bilayer could provide more space to locate a nitroxyl group [40]. Therefore, possible preferential presence of the nitroxyl group of 16-DS in the membrane environment where hydrophobic acyl chains of lipids fill the space more loosely might make the nitroxyl moiety more easily accessible for membrane reductors in comparison to Met-12-DS. Ubiquinol in membranes other than mitochondrial, including plasma membrane, has been also indicated to play an antioxidative role. Some amounts of ubiquinol were found to reside in the centre of the lipid bilayer and emerge into the other parts of the bilayer [47]. A reduced benzoquinone ring of ubiquinol was found to display a motion in a wide spatial range within the membrane between its extra- and intracellular surfaces [41]. Furthermore, in dimyristoylphosphatidylcholine (DMPC) multilamellar vesicles ubiquinol was able to interfere with hydrophobic acyl chains in less ordered phospholipid segments, and it could also slightly disorder the hydrophobic part of the membrane [47]. These results point to the possibility of more easy interference of ubiquinol with 16-DS than Met-12-DS, and hence, it can be assumed that 16-DS doxyl residues could be reduced faster, which could explain the higher rate of its reduction measured in our study than those of Met-12-DS. Since 16-DS inhibited LPO at 1000 μM concentration, we can suggest that oxidative reactions might have limited influence on the cytotoxicity of this nitroxide, and other membrane-associated mechanisms could have an impact.

Besides the presence of cellular reductors able to reduce a nitroxide, another factor, which might influence the rate of reduction is endocytosis. For instance, in the work performed on RBL-2H3 mast cells, the EPR signal of PC spin probes at 5 °C lowered in time according to monoexponential kinetics, but under these temperature conditions, nitroxide reduction is slower, because endocytosis is negligible [40]. It was simultaneously suggested that at higher temperatures a membrane spin probe could be reduced much faster due to the occurrence of endocytosis [40]. Additionally, in various cell types, which are characterized by high metabolic activity, including mast cells, metabolism of arachidonic acid (AA) by lipoxygenases (LOX) and cyclooxygenases (COX) also plays a key role in the production of ROS. Generation of ROS may lead to the fast reduction of doxyl nitroxides present in the membrane environment. However, for all DSs used in our EPR measurements performed at 37 °C, we observed a decay of the EPR signal intensity according to a linear function, which implies that the occurrence of endocytosis in B14 fibroblasts is rather less possible and might make its influence on nitroxide reduction practically insignificant.

In our investigation, one of the probes, i.e., Met-12-DS had an esterified carboxyl group (Figure 1B), which could suggest its different action and toxicity towards cells in comparison to the probes with non-esterified carboxyl group (5-DS and 16-DS; Figure 1A,C). Furthermore, Vogel and co-workers reported the effect of flexibility of the spin-labelled acyl chain and similar location of the nitroxide moieties within the membrane for the PS-10-doxyl-PC and PS-16-doxyl-PC phospholipid analogue EPR probes incorporated to 1-palmitoyl-2-oleoyl-sn-glycero-3-phosphocholine (POPC) membranes [48], which, consequently, could suggest analogous behaviour for Met-12-DS and 16-DS in the cell membrane.

However, a cellular lipid bilayer containing cholesterol and membrane proteins is much more complex than POPC membranes, and a carboxyl group or esterified carboxyl group present in the molecule of a probe anchors both forms in the surface part of the membrane. Additionally, the EPR spectra of Met-12-DS and 16-DS incorporated into fibroblast plasma membrane, in general, are similar, unlike in other cells. Hence, such a non-esterified or esterified carboxyl group might have limited influence on biological properties of the probe, and the difference in toxicity towards B14 fibroblast cells between Met-12-DS and 16-DS is unlikely to be due to its presence. Therefore, the other reason(s) for different cell toxicity shown in the trypan blue exclusion assay between Met-12-DS and 16-DS should be considered.

The EPR spectra of Met-12-DS and 16-DS incorporated into various membranes can also show some differences caused mainly by other location of a paramagnetic group (nitroxyl moiety) in the molecules of both probes, i.e., at different depths of the lipid bilayer of the membrane. On one hand, a membrane has a high degree of molecular disorder due to thermal motions, which undergo its molecules including incorporated probes causing a broad distribution profile of a probe in the POPC membrane, but simultaneously, the distribution of the nitroxyl moiety in the POPC membrane reaches a maximum characteristic for a particular probe [48]. The maximum for distribution of the probe with a nitroxyl group at the C-5 position (PS-5-doxyl-PC) indicates for location of the nitroxyl moiety mostly in the upper chain, glycerol, and headgroup parts of the POPC bilayer. For PS-10-doxyl-PC the maximum of distribution in the membrane locates this probe in the upper chain bilayer parts as well, however, with a shift more to the centre of the leaflet. Only for PS-16-doxyl-PC the maximum of distribution is shifted towards the lower chain part of the POPC membrane [48]. Simultaneously, it could be taken into account that spin-labelled phospholipids and doxyl stearates (spin-labelled derivatives of a fatty acid; Figure 1) might have different maximum of distribution in the membrane and, therefore, should locate a nitroxyl group with a relative shift at different depths of the lipid bilayer, because they are probes of various type. Nevertheless, based on the report of Vogel and co-workers indicating the existence of a maximum for a distribution of nitroxyl group of spin labels in the bilayer leaflet [48], in our study, we could expect the nitroxyl moiety of Met-12-DS in the region rich in double bonds of unsaturated fatty acids of lipids. This assumption was confirmed in our investigation by interactions of Met-12-DS with 12-AS fluorescent probe in the measurements of lipid membrane fluidity. Further, the nitroxyl moiety of 16-DS should have the maximum of distribution at the depth of saturated hydrocarbon chain fragments, and its motion is almost isotropic, while the 12-DS probe shows more anisotropic motion. Analogous differences were recorded for probes 12PC and 16PC at 5 °C. On the other hand, at 37 °C, the disappearance of anisotropic motion was detected; spectra of both probes were very similar and represented their isotropic motion [40].

Furthermore, Swamy and co-workers have indicated that PC EPR probes are able to partition both into more ordered and more disordered membrane domains, but simultaneously, they suggested that 5PC has a higher propensity to locate mainly in more ordered region of cellular membrane than, e.g., 12PC [40]. Based on these results [40] and also on the previous report that phospholipids and free fatty acids do not vary regarding the distribution width in a membrane of an attached doxyl group [48], we could suggest that the DSs might locate their nitroxyl moiety, in general, in similar regions of the cell membrane analogously to spin-labelled phospholipid derivatives in terms of both their position in relation to the membrane normal and lateral distribution. Therefore, 5-DS could locate in the plasma membrane of B14 cells preferably in its more ordered regions and could influence the function of “membrane rafts (MR)”. More ordered regions of the plasma membrane, referred to commonly as “lipid rafts” or “membrane rafts”, are known to incorporate different membrane proteins. These proteins, among them many membrane receptors, are involved in a number of crucial processes, including cell signalling and induction of cell death [49]. Since domains of ordered lipids in the plasma membrane of live cells have been shown to constitute a major part of lipid phase [40], 5-DS could possibly perturb cellular signalling and induce cell death to a higher extent than the other investigated DSs. Incorporation and the presence of the nitroxyl group of 5-DS both near the cell surface and in more ordered membrane regions where many membrane receptors and other important proteins are present [49] might at least partially explain the higher cytotoxicity of this nitroxide in comparison to Met-12-DS and 16-DS. The maximum of distribution of PS-10-doxyl-PC in the upper chain bilayer part in the membrane is shifted more to the centre of the leaflet compared to PS-5-doxyl-PC [48]. Hence, we could suggest that, used by us, Met-12-DS locates mainly in the central region of the leaflet, and the maximum of distribution of the nitroxyl group could not be present so close to the membrane surface as in the case of 5-DS [48]. Moreover, 10PC and 12PC were shown to locate in more ordered fragments of cellular membranes less preferentially than 5PC [40]. Therefore, we could suggest that Met-12-DS might locate mainly in a less ordered membrane environment in comparison to 5-DS and that Met-12-DS could influence membrane signalling and induce cell death to a lesser extent than 5-DS, which would be in agreement with our results. Further, 16PC has been shown to partition both into more and less ordered membrane phases with a slight preference for less ordered regions [40]. Therefore, we could suggest that preferential incorporation of 16-DS into more disordered parts of the membrane bilayer also might not be strong. However, in comparison to Met-12-DS, accumulation in the membrane of 16-DS with its nitroxyl group close to the central part of the bilayer along with quicker reduction could contribute to a lesser influence of this probe on cell signalling, and induction of cell death might take place after a longer time than in the case of Met-12-DS, which would be consistent with our results. This would particularly explain the difference in cell toxicity between Met-12-DS and 16-DS detected by us using two different methods, i.e., trypan blue exclusion assay and fluorescent double staining (Figure 2, Figure 3 and Figure 4).

Doxorubicin molecule locates mainly at the membrane surface between phospholipid headgroups [50], but its aglycone part can position in the deeper part of lipid leaflet [51], which enables oxidative reactions of DOX in hydrophobic environment and initiation of LPO [13]. An average 30% increase in LPO found in cells treated with 10 μM DOX in the present study, which is the concentration much larger than LC50 of this drug in B14 cells (0.5 μM), is in a good agreement with oxidative mode of action of DOX and our earlier results [29]. On the other hand, in the same study, we did not observe any significant increase in LPO after administration of LC50 concentration of the drug [29]. This might suggest that the fluidisation of cell membranes observed in this study was probably not caused by LPO, but rather by other more subtle changes in the structure of lipid bilayer, which could be detected fluorimetrically, and that LPO is not always a prerequisite for induction of changes in cell membranes. Relatively low level of cells undergoing cell death after their treatment with LC50 concentration of DOX and 24 h post-incubation in a drug-free medium is in accordance with earlier studies performed on B14 cells, which required much higher concentrations of DOX than LC50 and longer post-treatment incubation in cell medium for induction of apoptosis [52]. In addition to oxidative actions towards lipids, in the membrane, DOX is also able to oxidize proteins [11] and can deteriorate membrane antioxidant system involving protein free thiol groups.

### Effects of the Doxyl Stearate Nitroxides and α-Tocopherol in Combination with DOX

Natural saturated fatty acids (stearic, myristic, and particularly, palmitic) have been found to significantly inhibit in vitro cellular response to DOX-induced DNA damage in primary mouse embryonic fibroblasts and osteoblasts, but not in immortalised murine and in human cancer cells. DNA damage response is an important mechanism preventing cancer development, since it triggers a cascade of signalling events leading to cell cycle arrest or apoptosis. In this regard, natural saturated fatty acids showed a negative role in regulation of the pathways of DNA damage response, which might increase the rate of cell transformation, proliferation of cancer cells, and drug resistance [53]. Simultaneously, so far, the influence of various DSs applied together with DOX on the cell toxicity has not yet been investigated in detail. In some studies, DSs were used as tools in EPR technique, e.g., 5-DS and 12-DS were utilized to measure changes in lipid membrane fluidity induced by DOX in isolated rat cardiac mitochondria [54]. However, our study revealed that spin-labelled stearic acids have the potential to act differently than natural fatty acids and induce apoptosis in spontaneously immortalised B14 cells.

Although 5-DS in the concentration range of 10–200 μM reversed changes in membrane fluidity induced by 0.5 μM DOX, the nitroxide induced a significant increase in the incidence of cell death, even in cells incubated with a low concentration of 5-DS (10 μM) and 0.5 μM DOX, compared to the percentage of cell death in cells treated with DOX or a nitroxide only. The prevalent fraction of cells after incubation with a combination of 200 μM 5-DS and 0.5 μM DOX consisted of late apoptotic cells. Since DOX at concentrations lower than 10 μM did not increase LPO in B14 cells [29] and preincubation of cells with high concentrations of 5-DS (500 and 1000 μM) reduced LPO evoked by 10 μM DOX, we can suggest that LPO was not a crucial factor for induction of cellular toxicity by a combination of DOX with 5-DS.

Met-12-DS at 1000 μM concentration, similarly to α-Tocopherol, effectively inhibited LPO generated by 10 μM DOX. This effect, however, had no influence on the toxicity of Met-12-DS. Although Met-12-DS caused more pronounced changes in membrane fluidity, its toxicity in combination with DOX was lesser than toxicity of combination of 5-DS with DOX. However, we can judge based only on the results for the surface region of membrane bilayer, because, as in the case of Met-12-DS applied alone, we could not detect any changes in anisotropy due to quenching of 12-AS fluorescence.

Similarly to 5-DS, 16-DS at high concentration (1000 μM) reduced LPO to the level of control. A concentration of 100 μM of 16-DS provided protection against DOX cytotoxicity as measured using trypan blue exclusion assay and a combination of 16-DS with DOX was less cytotoxic than an anticancer drug applied alone, which could be related to the normalisation of fluidity in the hydrophobic part of the plasma membrane to the level of control. On the other hand, cellular changes were delayed, since the same concentration (100 μM) of nitroxide in combination with 200-fold lower concentration of DOX (0.5 μM) induced apoptosis in higher percent of cells than 16-DS alone. Concentrations of 1000 and 2000 μM of 16-DS combined with DOX, in general, induced cell death almost in all cells, comparably to 16-DS alone. Most of the cells incubated with 1000 μM 16-DS alone were in late apoptosis, while the highest concentration of nitroxide (2000 μM) caused necrosis in all cells. Unlike 5-DS, Met-12-DS, and α-Tocopherol, high concentrations of 16-DS (1000 and 2000 μM) combined with DOX caused necrosis in a considerably smaller fraction of the cells than 16-DS alone. Furthermore, 1000 μM 16-DS combined with DOX triggered late apoptosis in more cells than Met-12-DS, but in fewer cells than α-Tocopherol. The highest 2000 μM concentration of 16-DS in combination with DOX caused a much smaller decrease in cell viability than the same concentration of Met-12-DS and had the effect comparable to α-Tocopherol.

Collectively, although the investigated compounds at their higher concentrations decreased LPO induced by DOX and this effect did not protect from toxicity displayed towards cells, we can conclude that high concentrations of the compounds could prevent from initiation or could interrupt LPO triggered by DOX in membranes giving an antioxidative effect, while lower concentrations were ineffective in protection both at the membrane and whole cell [55]. Efficient inhibition of LPO by α-Tocopherol requires the presence of vitamin C, which is able to reduce the tocopheroxyl radical back to α-Tocopherol [56]. Therefore, efficiency of α-Tocopherol in protection against excessive LPO, e.g., evoked by a xenobiotic such as DOX, can be limited. Α-Tocopherol also induced weaker pronounced changes in membrane fluidity and was the less cytotoxic in our study. This compound is known to have a stabilising effect on membrane structure [27]. DSs locate in cell membrane similarly to phospholipid molecules and at different depths of their hydrocarbon chain they have a nitroxyl group, which determines their antioxidant properties and gives them the opportunity to inhibit LPO [55]. On the other hand, lower nitroxide concentrations might be insufficient to protect cell membranes [29]. Insufficiency in protection of plasma membranes by lower DSs concentrations might be related to nitroxide reduction to a corresponding hydroxylamine, which progressively decreased a DS concentration in time. Hydroxylamines of different nitroxides have been shown to possess cell protective properties, e.g., towards cardiomyocytes [20]. However, little is known on the protective or cytotoxic effects of hydroxylamines of EPR membrane probes, especially of DSs, towards cells. Our study, in general, revealed that cytotoxicity of the investigated DSs was only partially linked to the rate of their reduction. Although all of the used DSs influenced the toxicity of DOX, the changes in membrane fluidity induced by lower concentrations of the investigated compounds did not lead to an enhancement of the toxicity of 0.5 μM DOX, but higher concentrations induced an opposite effect acting synergistically with this anticancer drug. Only 100 μM 16-DS provided protection against the toxicity of DOX comparable to 100 μM α-Tocopherol. The fastest reduced nitroxide 5-DS induced the strongest augmentation of DOX toxicity in comparison to the drug alone. Taken together the results obtained for 5-DS, this nitroxide can be regarded as the strongest enhancer of DOX action in B14 cells among the three investigated DSs. The nitroxide reduced at an intermediate rate, i.e., 16-DS, at the highest concentrations in combination with DOX influenced cell viability weaker than Met-12-DS and displayed different effects on induction of late apoptosis and necrosis than other investigated compounds, especially Met-12-DS. For nitroxides other than spin-labelled fatty acids, especially piperidine derivatives, it has been shown that the rate of nitroxide reduction is dependent on several various factors including the presence and type of ring substituents [29]. In the case of DSs, however, other factors may play a role, such as order of lipids in a membrane region where a DS incorporates and the depth at which a nitroxyl group resides in the outer membrane leaflet. In the current study, albeit Met-12-DS was reduced the most slowly, simultaneously it was not the least cytotoxic. Therefore, the rate of reduction of the nitroxyl moiety does not seem itself to condition the properties of a nitroxide, but crucial could instead be the depth at which the nitroxyl group is present and the state of local environment in the cell membrane. Furthermore, mixed results obtained for different nitroxides from the measurements of changes in cellular membranes do not provide a straightforward explanation of the induced toxicity towards B14 cells. However, there are reports showing that 5-DS and 12-DS are able to undergo flip-flop translocation in membranes faster than 16-DS [57], and therefore, 5-DS and 12-DS could induce more perturbations in membrane structure and possibly in its asymmetry, which is known to be associated with apoptosis [58]. These results point to the possibility of the involvement of the mechanisms of the maintenance of cell survival or induction of its death based on the direct influence on plasma membrane physiological balance. In addition, the explanation of the effect of normalisation of the fluidity of the cell membrane observed in most cases for the intermediate concentrations of the investigated compounds, independently whether they were incubated in cells with DOX or not, is not straightforward. It is known that the effects of different compounds can be enhanced or diminished depending on their doses and molar ratio. Concentrations of the investigated compounds lower and higher than the intermediate could introduce some perturbations in the ratio of different lipids and membrane structure seen in the results of the measurements of lipid fluidity, while intermediate concentrations, mostly 50–100 μM, apparently induced a stabilising effect on the membrane structure. The previous study, conducted on MCF-7 breast cancer cells, revealed that the effects of two anticancer drugs, DOX and hydrophobic taxane paclitaxel, administered simultaneously, were completely different from their effects as single compounds and strongly depended on their concentration and, what is even more important, on the molar ratio of both drugs [5]. In this study, although DOX was present in the membrane at a much lesser concentration than DSs, its oxidative action could lower the amount of protein free thiol groups, which might influence the rate of nitroxide reduction causing elongation of the times of DS half-reduction. Moreover, the effect of DOX was more pronounced towards the fastest reduced 5-DS (about 30% of prolongation of half-reduction time in the presence of DOX in comparison to the nitroxide alone) than for 16-DS (half-reduction time longer for about 25%) and especially towards the slowest reduced Met-12-DS (approx. 15% longer half-reduction time) (Table 1B). Therefore, it can be concluded that the closer to the membrane surface (5-DS), or if lipids were less densely packed (16-DS) in the outer leaflet, then the effect of a combined treatment of cells with DOX and a nitroxide was more evident.

It is well known that in the case of many compounds, the time required to detect changes induced in cells should be much longer than the time of incubation. For instance, in the previous study, after a 2 h treatment of B14 cells with 1 μM DOX, further culturing of cells for 24 h or longer in a drug-free medium was required to detect DNA fragmentation using agarose gel electrophoresis of DNA [52]. Therefore, we can suggest that the effect of the compounds investigated in the current study in cells was similarly prolonged in time, giving the difference in the results between the trypan blue exclusion test and other evaluations performed after 24 h.

## 4. Experimental Section

### 4.1. Materials

#### 4.1.1. Chemicals

Doxorubicin (Adriamycin) was purchased from Sequoia Research Products Ltd. (Pangbourne, UK). The following reagents: 5-doxyl-stearic acid [2-(3-Carboxypropyl)-4,4-dimethyl-2-tridecyl-3-oxazolidinyloxy] (5-DS; Figure 1A), 16-doxyl-stearic acid [2-(14-Carboxytetradecyl)-2-ethyl-4,4-dimethyl-3-oxazolidinyloxy] (16-DS; Figure 1C), α-Tocopherol (DL-all-*rac*-α-Tocopherol), fluorimetric probes 12-AS [12-(9-anthroyloxy)-stearic acid] and TMA-DPH (1-[4-trimethylammoniumphenyl]-6-phenyl-1,3,5-hexatriene), dimethyl sulfoxide (DMSO), ferrous sulfate, deoxyribose (DR), trypan blue, propidium iodide (PI), Hoechst 33258 (2-[2-(4-hydroxyphenyl)-6-benzimidazoyl]-6-(1-methyl-4-piperazyl)benzimidazole trihydrochloride) and Tris were purchased from Sigma (Sigma-Aldrich, St. Louis, MO, USA). Sodium dodecyl sulfate (SDS) was obtained from Merck (Darmstadt, Germany). Methyl 12-doxyl-stearate (Met-12-DS; Figure 1B) was synthesized according to Waggoner method [59]. Chromium(VI) oxide was used to oxidize 12-hydroxyl-stearic acid. Oxazolidine ring was attached to methyl stearate using 2-amino-2-methyl-1-propanol, and amine was oxidized to nitroxide with 3-chloroperbenzoic acid. We decided not to perform hydrolysis of the methyl ester to the carboxylic acid based on indications from the previous studies using 12-DS and 12-DS methyl ester spin probes on various cells, including fibroblasts, which have shown that the use of methyl ester enables for obtaining a better EPR spectrum of the probe incorporated into the membrane [4]. Since the use of Met-12-DS improves the sensitivity of EPR measurements, we decided to perform our current investigation on the cytotoxic effects of doxyl nitroxides using this spin probe and not 12-DS. Methyl ester of 12-doxylstearic acid, which is a viscous yellow oil, was purified by a preparative thin layer chromatography using hexan-ether (7:3). IR spectroscopy was applied to detect spectrum of Met-12-DS in chloroform, and a typical band for ester carbonyl was found at 1730 cm^−1^.

All other chemicals, i.e., ethyl alcohol anhydrous 99.8%, hydrochloric acid (HCl), hydrogen peroxide (H_2_O_2_), potassium dihydrogen phosphate, di-potassium hydrogen phosphate, sodium hydroxide (NaOH), 2-thiobarbituric acid (TBA), and trichloroacetic acid (TCA) were purchased from POCH S.A. (Gliwice, Poland) and were of the highest analytical grade available. Deionized Q water (Millipore Corp., Bedford, MA, USA) was used in the preparation of all solutions except the TBARS assay where double distilled water was utilized.

#### 4.1.2. Cell Culture

Fibroblasts from the peritoneum of Chinese hamster (cells, which underwent spontaneous immortalisation, B14 cell line) were obtained from The Central Cell Bank of the Child Health Center (Warsaw, Poland). Nunclon (Roskilde, Denmark) was a supplier of cell culture flasks and dishes. Eagle’s minimal essential medium (MEM), trypsine, and phosphate buffered saline (PBS) were obtained from BioMed (Lublin, Poland). Foetal calf serum (FCS) was supplied by Gibco (Invitrogen, Edinburgh, Scotland).

Monolayer cultures of cells were kept under optimal conditions: 37 °C, 100% humidity, 5% CO_2_. MEM enriched with 10% FCS was used as a growth medium of cells, which were subcultured every 2–3 days for their maintenance in the exponential growth phase. Routine monitoring of cultures was performed to exclude *Mycoplasma* contamination.

### 4.2. Methods

#### 4.2.1. Incubation of Cells with the Investigated Compounds

Before experiments, cells were plated out on Petri dishes or in cell culture flasks for subculturing. The cells were grown for at least 16 h before the incubation with the investigated compounds to ensure that they were in the exponential phase of growth. Except EPR measurements (described in the Section 4.2.6), in all other assays, the same pattern of cell treatment under culture conditions was employed: incubation with a nitroxide or α-Tocopherol alone for 3 h, or incubation with DOX alone for 2 h. In experiments with combined treatment, cells were first incubated with a nitroxide or α-Tocopherol for 1 h, and next, DOX was added and incubation was continued for an additional 2 h. In the previous experiments, LC50 for DOX in B14 cells was determined as 0.5 μM concentration [29]. Therefore, in this study, we used 0.5 μM DOX for cell viability assay, assessment of cell morphology, determination of cell death, and membrane fluidity measurements. Only in the TBARS assay 10 μM concentration of DOX was used. Control cells were incubated with PBS for 2 h (treatment with DOX only) or 3 h (treatment with α-Tocopherol, 5-DS, Met-12-DS, and 16-DS alone or in combination with DOX).

#### 4.2.2. Determination of Cell Viability by Trypan Blue Exclusion Test

B14 cells were trypsinized and incubated with the investigated compounds as described above. After incubation, cells were washed twice with PBS and immediately (0 h time point) stained with trypan blue (final concentration of the dye 0.2%). All cells and blue-stained dead cells were counted with an Olympus IX70 microscope (Tokyo, Japan) and the percentage of viable and dead cells was calculated in relation to all cells (100%) [60].

#### 4.2.3. Evaluation of Apoptotic and Necrotic Cell Death by Double Staining Technique

The effects of the investigated compounds on the condition of cells were assessed by estimation of cell morphology and changes associated with apoptosis/necrosis after double staining of cells with fluorescent dyes Hoechst 33258 and PI, allowing for detection of apoptosis and necrosis. The cells were incubated with the investigated compounds, as described above; however, the concentrations of 5-DS and Met-12-DS were chosen in a narrower range than for α-Tocopherol and 16-DS (Figure 3A,B) based on the results from measurements of cell viability using trypan blue (Figure 2). After incubations, the cells were washed once with PBS and cultured in a fresh medium for further 24 h. After 24 h of growth, the culture medium was replaced with Hanks’ balanced salt solution (HBSS), and morphology of B14 cells was analysed using an Olympus IX70 microscope (Tokyo, Japan) under magnification 150×. For double staining, Hoechst 33258 to the final concentration of 0.13 mM and PI to the final concentration of 0.23 mM were added. Then, the incubation of cells with the dyes was carried out for 10 min (room temperature, in the dark). After incubation, cell monolayers were analyzed using an Olympus IX70 microscope under magnification 150×. The cellular changes, visualized with the two dyes Hoechst 33258 and PI, allowed for classification of the cells as viable (blue fluorescence), early apoptotic (intensive bright blue fluorescence), late apoptotic (blue-violet fluorescence), and necrotic (red fluorescence) [61]. Three independent experiments were performed, each in three repeats, and the percentage of each type of cells was calculated in relation to the total number of cells (100%).

#### 4.2.4. Plasma Membrane Fluidity Measurements

Fluorescence spectroscopy was employed to evaluate changes in fluidity of the plasma membrane of B14 cells induced by the investigated compounds. Plasma membrane fluidity was estimated on the basis of fluorescence anisotropy values measured for TMA-DPH and 12-AS fluorescent probes. TMA-DPH is a cationic fluorescent aromatic hydrocarbon, which mainly incorporates at the lipid–water interface and probes the surface polar region of the plasma membrane. The fluorescent probe 12-AS localises deeper in the lipid bilayer and probes the hydrophobic core of the membrane. Fluorescence anisotropy of a molecule of the fluorescent probe depends on its free rotation in the lipid layer, which is inversely related to the plasma membrane fluidity. An increase in membrane fluidity is generally linked to a decrease in lipid order, which causes a decrease in anisotropy of the fluorescent probe. Conversely, a decrease in membrane fluidity is reflected by an increase in lipid order and an increase in fluorescence anisotropy. We have chosen these probes to check how the investigated compounds influence on both the surface (polar) and hydrophobic regions of the plasma membrane.

B14 fibroblasts suspended in Tris-KCl buffer (pH 7.4), at a density of 4 × 10^5^ cells/mL, were incubated with TMA-DPH or 12-AS at 1 μM final concentration for 4 min (TMA-DPH) or 10 min (12-AS) at 20 °C in the dark. The intensity of fluorescence was measured on PerkinElmer Luminescence Spectrometer LS50B. The following wavelengths were set: 360 nm or 365 nm for excitation of TMA-DPH or 12-AS, respectively, and fluorescence emission was measured at 425 nm for TMA-DPH or at 471 nm for 12-AS. The fluorescence anisotropy (r) was obtained from the equation:

r = 2P/(3 − P)(1)

where P = (Ivv − IvhIhv/Ihh)/(Ivv + IvhIhv/Ihh), Ivv is the intensity of parallel emission, and Ivh is the intensity of perpendicular emission, in relation to the direction of excitation light polarization (P) [62].

#### 4.2.5. The TBARS Assay

For measurements of the TBARS content the standard method [63] was used with modifications. Cells were suspended at the density of 1.6 × 10^6^/mL in PBS, and then the investigated compounds were added. Following incubation and centrifugation, cells were washed twice with PBS, and double distilled water was added. Cell lysis was performed by freezing at −20 °C. Since untreated (control) cells gave very small amounts of colour malondialdehyde (MDA) product, we modified the protocol by adding DR, H_2_O_2_, and Fe (II) solutions to all samples in order to obtain a more intense colour and measurable MDA absorbance. Appropriate solutions were added to the cell lysates to achieve the final concentrations of 0.81% SDS, 5 mM DR, 50 μM H_2_O_2_, 50 μM Fe(II), potassium phosphate buffer (pH 7.4). Then, incubations for 30 min at 20 °C and after the addition of 2.8% TCA and 1% TBA in 50 mM NaOH, for 20 min at 100 °C were performed. At the end of incubations, samples were put on ice to precipitate SDS and proteins, and centrifuged. The absorbance of MDA was measured at 532 nm on CARY 50 Bio spectrophotometer (Varian Inc., Mulgrave, Australia) against a blank sample containing all reagents except cell lysate and DR. The molar extinction coefficient for MDA (ε _532 nm_ = 1.56 × 10^5^ M^−1^ cm^−1^) was used to calculate the concentration of TBARS, which was finally expressed as a percentage of control (100%).

#### 4.2.6. EPR Measurements

EPR measurements were carried out at X band (9.6 GHz of microwave frequency) using ESP-300E Bruker spectrometer set at 20 mW of microwave power, 338 mT of the central field, 10 mT of the sweep width, and 100 μT of the field modulation amplitude. All experiments were performed on samples prepared in EPR-silent quartz glass capillaries kept in a water bath at 37 °C between the measurements.

Investigations of the stability of each of the used DSs were performed using ethanol solutions of these compounds added to 80 μM final concentration. Interactions with DOX were checked after mixing of ethanol solutions both of a nitroxide and DOX to 80 μM final concentration. To measure the stability of the DSs and their possible interactions with DOX the spectrometer was set as follows: 64 s sweep time, 0.3 s time constant, and 10^4^ receiver gain, and the measurements were done directly after mixing of solutions, 3 h and 24 h later.

Although in EPR technique in most cases for spin-labelling of cells, the standard 100 μM concentration of a nitroxide is used, in our measurements of reduction of DSs in B14 cells, we applied experimentally established conditions. To obtain the most optimal signal to noise ratio and, therefore, the best spectra as possible, measurements were performed for final 80 μM concentration of the DSs and with the use of the spectrometer set as follows: 256 s sweep time, 1 s time constant, and 10^5^ receiver gain. These experiments were done using B14 cells suspended in HBSS at the density of 15 × 10^6^ of cells/mL. Directly before the measurements, a DS was added to cells to obtain its final 80 μM concentration in 100 μL of the cell suspension, and immediately the suspension was mixed and vortexed for 40 min in order to incorporate a DS into the cell membrane. After vortexing, the cell suspensions were centrifuged, the supernatants were discarded, and the cell pellets were resuspended in 50 μL of fresh HBSS, the suspensions were placed in EPR capillaries and the EPR measurements were immediately started and repeated 4 to 6 times in 30–40 min intervals, until the EPR signal intensity was at least below 50% of its initial value.

The influence of DOX on reduction of the DSs was checked in parallel experiments, in which DOX dissolved in HBSS was added to cells to 1 μM final concentration directly before the addition of a DS.

It is known that EPR signal intensity of a paramagnetic probe depends on its concentration. Therefore, we analysed the height of the middle-field line (*h*_0_) of the EPR spectra of the DSs. For each sample, the *h*_0_ value obtained from an initial measurement was considered as 100% of the EPR signal intensity. Values of *h*_0_ obtained from further measurements were recalculated into the percent of the EPR signal intensity in relation to an initial value from the equation of a linear function:

% *h*_0 *t*_ = *k* × *t* + % *h*_0 *t*=0_ (2)

where: % *h*_0 *t*_—percent value of the height of the middle-field line (*h*_0_) of the EPR spectrum of a DS at specific time point different from zero; *k*—nitroxide reduction rate constant; *t*—time [min]; % *h*_0 *t=*0_—100% of the height of the middle-field line at time point zero, which is the time of the first measurement.

Based on the Equation (2), the rates of reduction of the DSs in B14 cells as well as the time of half-reduction (*t*_½_) of the probes were calculated.

#### 4.2.7. Statistical Analysis

All measurements were carried out at least in three independent experiments. The data were shown as a mean ± standard deviation (SD). The normality of the data distribution was checked using the Shapiro–Wilk test. The homogeneity of variance was assessed by the Levene’s test. For the dataset with homogenous variances, the statistical difference between groups was evaluated by ANOVA, and the significance of differences was determined using the post hoc Tukey’s test. For the EPR measurements, to check the statistical significance for comparisons of a DS with a DS+DOX, we used Student’s t test for independent data. When variances were non-homogenous, we applied a non-parametric Kruskal–Wallis test and the post hoc Conover–Inman test. Statistical significance for comparisons of a DS with a DS+DOX was assessed using the Mann–Whitney U test (EPR data). Calculations were performed employing the STATISTICA (2000, StatSoft Inc., Tulsa, OK, USA) and Stats Direct software (StatsDirect Ltd., Merseyside, UK).

## 5. Conclusions

In conclusion, the results of this study indicate that changes in membrane fluidity and toxicity, caused by doxyl nitroxides 5-DS, Met-12-DS, and 16-DS, common spin labels used in the EPR technique, as well as the rate of their reduction to EPR-silent hydroxylamines should be taken into consideration in different biophysical investigations. These nitroxides should be used more carefully as paramagnetic probes for measuring the influence of other compounds on the state of the membrane bilayer in specific types of cells with susceptible membranes, such as in B14 cells. Furthermore, the results presented herein point to further in vitro studies on human cancer cell lines and possibly to in vivo studies, since DS compounds acted promisingly and, unlike natural stearic acid, showed their potential as sensitizers of cancer cells to the actions of an anthracycline anticancer drug doxorubicin.

## Figures and Tables

**Table 1:** (**A**) The rates of reduction (k) in B14 cells of the doxyl stearate nitroxides (5-DS, Met-12-DS, and 16-DS) applied alone at the final concentration of 80 μM or together with 1 μM doxorubicin (DOX). The data are presented as means ± SD of at least three independent experiments. (**B**) Half-reduction times in B14 cells of the doxyl stearate nitroxides (5-DS, Met-12-DS, and 16-DS) applied alone at the final concentration of 80 μM or together with 1 μM doxorubicin (DOX). The data are presented as means ± SD of at least three independent experiments.

**(A)**			
**Compound(s)**	**The Rate of Nitroxide Reduction (k) ± SD**	**Statistical Significance**	
5-DS	−0.7887 ± 0.0638	vs. other DSs applied alone	*p* < 0.05 vs. Met-12-DS
Met-12-DS	−0.5064 ± 0.1347		*p* < 0.05 vs. 5-DS
16-DS	−0.6970 ± 0.1334		n.s.
1 μM DOX + 5-DS	−0.649 ± 0.1138	vs. the same DS applied alone	n.s.
1 μM DOX + Met-12-DS	−0.4707 ± 0.1328		n.s.
1 μM DOX + 16-DS	−0.5877 ± 0.1082		n.s.
**(B)**			
**Compound(s)**	**Half-Reduction Time of a Nitroxide (t _½_) [min] ± SD**	**Statistical Significance**	
5-DS	63.66 ± 5.01	vs. other DSs applied alone	*p* < 0.01 vs. Met-12-DS
Met-12-DS	104.42 ± 26.04		*p* < 0.01 vs. 5-DS; *p* < 0.05 vs. 16-DS
16-DS	73.66 ± 15.19		*p* < 0.05 vs. Met-12-DS
1 μM DOX + 5-DS	78.82 ± 13.62	vs. the same DS applied alone	n.s.
1 μM DOX + Met-12-DS	113.52 ± 31.32		n.s.
1 μM DOX + 16-DS	87.34 ± 16.50		n.s.

n.s.—not significant.
